# Primary Breast Angiosarcoma: Avoiding a Common Trap

**DOI:** 10.1155/2011/517047

**Published:** 2011-07-06

**Authors:** Christine Desbiens, Jean-Charles Hogue, Yves Lévesque

**Affiliations:** ^1^Centre des Maladies du Sein Deschênes-Fabia, Hôpital du Saint-Sacrement, CHA Universitaire de Québec, 1050 chemin Sainte-Foy, Québec City, QC, Canada G1S 4L8; ^2^Santé des populations: URESP, Centre de recherche FRSQ du CHA Universitaire de Québec, 1050 chemin Ste-Foy, Quebec City, QC, Canada G1S 4L8; ^3^Département de Chirurgie, Université Laval, Pavillon Ferdinand-Vandry, 1050 Avenue de la Médecine, Quebec City, QC, Canada G1V 0A6; ^4^Hôtel-Dieu d'Arthabaska, 5 Rue des Hospitalières, Victoriaville, Québec City, QC, Canada G6P 6N2

## Abstract

*Background*. Primary breast angiosarcoma is a rare entity. *Case*. Initial diagnosis was a benign hemangioma at core biopsy. Wide local excision was performed, with positive margins. Pathology after surgery reported a moderately differentiated angiosarcoma. Tumor was finally treated using mastectomy and radiations. She developed a second angiosarcoma in contralateral breast, with an initial diagnosis on core biopsy of an atypical vascular lesion and was again treated using mastectomy and radiations. She developed bones and lung metastases. *Conclusion*. Primary breast angiosarcoma is a rare entity often difficult to diagnose on core biopsy, and a benign differential diagnosis is frequent. A highly vascular breast mass should always be considered malignant until proven otherwise. Surgical treatment seems to be the best course of action. There is a lack of data proving efficacy of adjuvant chemotherapy and radiation therapy.

## 1. Introduction

Primary breast sarcomas are rare entities. These malignant tumors originate from mesenchymental glandular breast tissue and account for <1% of all breast cancer cases [1]. The more frequent histological subtypes encountered are malignant fibrous histiocytoma, fibrosarcoma, liposarcoma, and angiosarcoma. Most angiosarcomas are secondary to radiotherapy treatments for breast cancer or to an arm lymphoedema subsequent to a modified radical mastectomy. Only about 20% of angiosarcomas are primary sarcomas. The incidence of primary breast angiosarcoma is about 17 new cases per million women. Most literature about these cases is case reports, and this tumors' natural history is thus only partially understood and its treatment lacks uniformity. The present case report presents a new case and reviews the existing literature in the aim of achieving a better definition of the treatment of this cancer and avoiding the common trap of a benign differential diagnosis.

## 2. Case Report

A 28-year-old patient, mother of two children, presented in October 2003 for a painless slowly growing mass in her left breast. She had no personal or family history of breast or ovarian cancer. Except for the suspect mass, she was in good health.

Since her last pregnancy in 2000, her left breast progressively enlarged. However, in the last 3 months, the enlargement rate increased, to the expense of the superior and median quadrants. She had no other breast symptom. Mammography showed a nonspecific and diffuse density area of about 7 cm and septa measuring up to 10 cm. There was no mammographic microcalcification or distortion nor any skin anomaly.

At physical examination, there was an important asymmetry at the expense of the left breast upper area. Repeated sonography showed a diffuse and ill delimited hyperechogenic infiltration in the superior portion of the left breast, spanning 16 cm in transversal and 10 cm in cephalocaudal planes. There was no infiltration of the pectoral muscle. A core needle biopsy (CNB) was performed and demonstrated the presence of a nonatypical vascular lesion interpreted as a benign capillary hemangioma.

We decided to try to remove the whole mass in November 2003. During surgery, in the lower portion of the left breast, margins were much ill defined and the lesion seemed more aggressive than a simple hemangioma. A sample of the tumor was sent to the pathology laboratory for intraoperative consultation, and the frozen section diagnosis was one of an “atypical vascular tumor, final diagnosis of benign or malignant tumor deferred on permanent section”. Hemostasis was also difficult to achieve, and surgical decision was to perform a wide local excision.

Final pathology diagnosis was a moderately differentiated angiosarcoma (grade II/III), measuring 12 cm on its larger axis. However, the tumor could be larger since all margins were positive. There was no skin invasion. Both estrogen and progesterone receptors were negative. Postsurgery blood screenings and axial tomography were all normal. We completed surgical treatment with a total left mastectomy extended to the skin in December 2003. A TRAM reconstruction was performed in order to replace removed skin and to close the wound.

**Figure 1:**
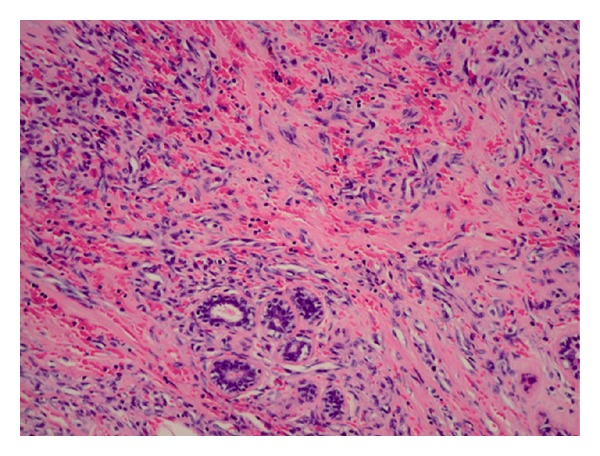
Hematoxylin and eosin stain on an ill-differentiated part of the left breast's angiosarcoma (200x).

**Figure 2:**
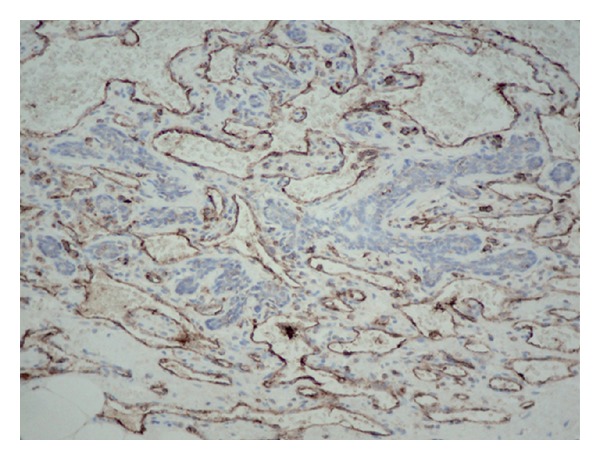
CD31 immunohistochemistry in left breast's angiosarcoma (200x).

Pathology on total left mastectomy confirmed a residual angiosarcoma grade II/III. Figure 1 shows an ill-differentiated part of the tumor (hematoxylin and eosin stain, 200x).  Figure 2 shows tumor positivity for CD31 (immunohistochemistry, 200x). All margins were finally negative. After tumor board discussion, no adjuvant chemotherapy was administered. She received radiation therapy to the left chest wall (60 Gy in 30 fractions) since local recurrence of this type of tumor was very likely. She received all her treatments, and she had a followup, including physical examination and CT scan, on a 6 months basis. In November 2004, there was no recurrence in the reconstructed left breast, and the right breast was normal. A cohesive gel implant was installed in the left breast in April 2005.

**Figure 3:**
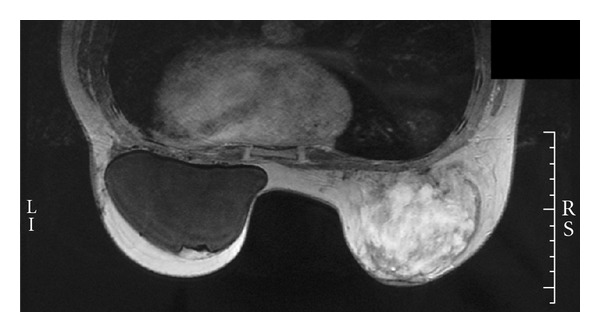
Magnetic resonance imagery showing the left breast implant and the highly vascular mass encompassing most of the right breast.

On December 2005, a suspect 2 cm mass was discovered in the right breast. Fine needle aspiration (FNA) was performed at her local medical centre and was negative. Further investigations using mammography, sonography, and CNB were performed and were reported as benign. Hyperechogenic area in lower part of the right breast was consistent with an hematoma. She was referred at our center in April 2006. Lesion was then 6 × 8 cm, well fixed to cutaneous tissue, but otherwise mobile. The hyperechogenic area was still present, with much ill defined borders. A sonography-guided NCB was performed, and material retrieved contained much blood. Pathology diagnosis was an atypical vascular lesion, and a well-differentiated angiosarcoma could not be ruled out. The magnetic resonance imagery (MRI) (Figure 3) showed the implant in the left breast and showed the mass in the right breast, encompassing most of the breast. Since it was impossible to rule out the possibility that it was a portion of a well-differentiated angiosarcoma, a total mastectomy with immediate reconstruction of the right breast was performed in April 2006. Pathology reported a well- to poorly-differentiated angiosarcoma (grade I/III to III/III) measuring 8 cm on its longer axis. Tumor was ill delimited with invasion of adipose tissue and mammary parenchyma. At the center of this mass, necrosis and blood lakes were found. There was dermal invasion, but epidermis was intact. Estrogen and progesterone receptors were negative. Endothelial cells bordering blood vessels were positive for CD34, CD31, factor VIII, and vimentin and were negative to keratin 8-18, concordant with a vascular origin. The 2006 tumor was compared to the one from 2003 and was very similar in their well-differentiated parts. One margin was positive and had to be removed in May 2006. A total radiation dose of 60 Gy was administrated in 30 fractions to the right chest wall. In November 2006, a PET scan showed some infiltrates in the right lung, suggestive of postirradiation changes.

In December 2008, a lytic lesion was found in the right humerus encompassing the whole cortex and the posterior portion of the humeral head. There was also an osteolytic lesion in the D8 vertebrae. The humeral lesion was metabolically highly active. The whole humeral head was removed and replaced by a prosthesis in January 2009. Pathology reported an angiosarcoma without invasion. The area was irradiated with a total dose of 20 Gy in 5 fractions. The vertebral lesion was treated using radiation (40 Gy in 15 fractions).

In July 2009, multiple bilateral lung metastases were found, and a progression was observed in the D8 lesion. Patient received palliative care.

## 3. Discussion

Breast angiosarcoma can be observed as a primary neoplasm or, more commonly, secondary to breast-conserving surgery combined to radiation therapy. In the present paper, in accordance to the case reported, primary angiosarcomas only will be discussed. Primary breast sarcomas and angiosarcomas represent, respectively, less than 1% and 0.04% of all breast cancers [1]. Breast angiosarcoma is more frequent in young women (20 to 50 years) with no previous cancer history or other known risk factors [2, 3]. Between 6 and 12% of primary breast angiosarcomas are diagnosed during pregnancy or shortly after, suggesting hormones involvement. However, cases reported to display positive estrogen receptors are so rare, that it is presently impossible to establish a link between angiosarcomas and hormonal dependency [4, 5]. In the case reported here, estrogen and progesterone receptors were both negatives.

In most published cases, breast angiosarcoma presents as a palpable mass, without pain and with a fast growing rate [6, 7]. Large or superficial tumors often present purplish, ecchymosis-like skin coloration [7]. In most cases, absence of pathognomonic characteristics specific to angiosarcomas will result in a wrong or delayed diagnosis [6, 8]. Radiologic characteristics may help to establish right diagnosis, but, most often, as in the present case, mammography is unspecific and heterogeneous. Sonography and MRI are useful in characterizing breast lesions, but again there is no distinctive features to angiosarcomas [6, 7]. MRI shows a low signal on T1-weighted images and a high signal on T2-weighted images [9, 10]. Positron emission tomography (PET) scan using 18F-FDG has shown an intense FDG accumulation in angiosarcomas and might be useful [9, 11].

In most cases, tumor size at diagnosis is larger than 4 cm [4]. As most soft-tissue sarcoma in most anatomical sites and of most histological subtype, angiosarcomas larger than 5 cm are associated to a shorter disease-free survival than angiosarcomas smaller than 5 cm. Indeed, tumors smaller than 5 cm are usually associated to a better prognosis, even in the presence of worsening factors. With small tumors, recurrence is usually local and distant metastases are infrequent. However, tumors larger than 5 cm are associated to distant metastases, independently of local recurrence [1, 12]. There is a need for a close follow-up, including computed tomography scans at 6-month intervals.

Sondenaa et al. conducted a survey of metastatic sites of all reported primary breast angiosarcoma. They concluded that the liver was the more frequent metastatic site, followed by lung, lymph nodes, bones, bone marrow and, less frequently, ovary, kidney, omentum, adrenal gland, stomach, pancreas, peritoneum, esophagus, and skin [13]. Breast angiosarcomas are stratified according to three grades. Well-differentiated tumors (grade I) are composed of anastomosing vascular channels that surround breast ducts and infiltrate the adipose tissue. Blood vessels are lined by a single layer of endothelial cells with hyperchromatic nuclei showing little mitosis. No endothelial tufting is seen. Well-differentiated breast angiosarcomas are associated to a longer recurrence-free survival and to fewer distant metastases. Moderately differentiated angiosarcomas (grade II) are largely like well-differentiated tumors but show small foci of solid proliferation of spindle-shaped cells and more mitotic figures. Poorly-differentiated angiosarcomas present more solid and atypical cell proliferation and often show necrotic area and blood lakes. Grade III tumors are associated to decreased five-year survival rate and to increased metastases rate [4].

Diagnosis prior to surgery, either by FNA or NCB, is at best difficult [4]. Chen et al. reported a percutaneous biopsy false-negative rate of 37% [14]. Differential diagnosis of this rare tumor include: benign hemangioma, cystosarcoma phyllodes, stromal sarcoma, metaplastic carcinoma, fibrosarcoma, liposarcoma, squamous cell carcinoma with sarcomatoid features, myoepithelioma, fibromatosis, and reactive spindle cell proliferative lesion [4, 15]. Large-core macrobiopsies might be useful to improve diagnosis prior to surgery since a larger sample is taken [16]. However, in the present case, the mass was so importantly hemorrhagic that a macrobiopsy would have been very difficult to perform. Surgical resection and microscopic examination of sufficient sampling of the tumor are often necessary to render a final diagnosis. Immunohistochemical examination (factor VIII and CD31 positivity) will confirm the vascular nature of the tumor [4, 7].

It is now well recognized that complete surgical excision of breast angiosarcoma is the best course of action and that total mastectomy is the best option [17]. Since hematogen dissemination is more than likely, axillary node dissection is not indicated [6, 15, 17, 18]. Also, many authors' opinion is that breast conservative surgery is not acceptable [6, 14, 19, 20], since the recurrence rate of wide local excision is 23% versus 8% for total mastectomy [21]. However, according to some authors, wide local excision would be indicated if the tumor is smaller than 5 cm [15, 22]. There is not enough data in the current literature to support either course of action. Also, since sarcomas are very active and invasive lesions, negative surgical margins are difficult to achieve at first surgery. In many reported cases, it is often said that surgical margins are positive after breast conservative surgery and that total mastectomy must then be performed [6, 14, 15], as in the present case.

It is generally recognized that breast angiosarcomas have a bad prognosis. However, prognosis depends upon tumor grade (most important factor), tumor size at diagnosis, and margin status at surgery [6]. Generally, 33% of patients with breast angiosarcoma, all grades together, are disease-free 5 years after initial diagnosis. Most of patients with a grade I/III tumor are alive after 15 years. Grade III/III tumors are the most aggressive, and the median disease-free survival is about 15 months. 

Chemotherapy and radiation therapy may be used as adjuvant treatment. However, the role of adjuvant chemotherapy is ill defined, because of the rarity of primary breast angiosarcoma and of the lack of prospective studies [6, 15, 22]. Most primary breast angiosarcomas are treated the same way as breast angiosarcoma secondary to radiation therapy. In most reported cases with use of chemotherapy, authors prescribed cyclophosphamide, anthracycline, or an alkylating agents combined to a pyrimidine analog [6]. Paclitaxel have been shown to produce excellent responses in a number of studies in a variety of anatomic sites [23–28]. New agents against angiogenesis, such as bevacizumab [29–33] or rapamycin [34], might also be useful against this tumor. Study by Sher et al. shows a 48% overall response rate to chemotherapy, especially using anthracycline-ifosfamide or gemcitabine-taxane therapies [5]. Chemotherapy might have a greater efficacy in higher grade tumors [15]. Concerning radiation therapy for breast angiosarcoma, data are insufficient and there is no clue about its efficacy to improve disease-free survival or to prevent metastatic spread. Also, no recovery was ever reported with the use of radiation therapy alone, and angiosarcomas seem to have some resistance to radiations [6]. Since the high likelihood of locoregional recurrence, radiation therapy might be indicated even after a total mastectomy [35]. Indeed, in the case reported here, the patient received radiation therapy to her left breast and she did not develop a recurrence to this breast. As with any cancer type, margins status is a major risk factor for recurrence [17]. It is why some authors recommend adjuvant radiation therapy when surgical margins are positive [36, 37] or when surgical margins are less than 2 cm [38]. Radiation therapy may be used for palliative treatment of pain secondary to bone metastasis [6]. As future therapy, targeted treatment against angiogenesis might be added to the therapeutic arsenal against angiosarcoma.

## 4. Conclusion

A large and metabolically active vascular mass in the breast should always be considered at first sight to be an angiosarcoma, until proven otherwise. Even if these tumors have a bad prognosis, surgical treatment using total mastectomy are preferred; wide local excision may be selected depending on tumor size. Early and precise diagnosis is an important prognostic factor. A close followup within the same centre is capital. Primary breast angiosarcoma is a rare entity, literature contains few data concerning adjuvant treatment, and there is no generally agreed course of action. Hormonal treatment doesn't seem to be appropriate since these tumors usually do not express estrogen receptors. Randomized, controlled, prospective studies should be conducted to have a better understanding of the role of adjuvant treatment in breast angiosarcoma. However, rarity of the disease might be a serious problem to these studies. It is thus important to report these cases and to present treatments and patient's evolution.
